# The Roles of Lactate and Lactylation in Diseases Related to Mitochondrial Dysfunction

**DOI:** 10.3390/ijms26157149

**Published:** 2025-07-24

**Authors:** Fei Ma, Wei Yu

**Affiliations:** 1 College of Life Sciences and Medicine, Zhejiang Sci-Tech University, Hangzhou 310018, China; 2024220902034@mails.zstu.edu.cn; 2 Zhejiang Provincial Key Laboratory of Silkworm Bioreactor and Biomedicine, Hangzhou 310018, China

**Keywords:** lactate, lactylation, epigenetics, mitochondrial dysfunction, metabolic reprogramming

## Abstract

Glycolysis and oxidative phosphorylation are the main pathways of cellular energy production. Glucose is metabolized via glycolysis to generate pyruvate, which, under anaerobic conditions, is converted into lactate, while, under aerobic conditions, pyruvate enters mitochondria for oxidative phosphorylation to produce more energy. Accordingly, mitochondrial dysfunction disrupts the energy balance. Lactate, historically perceived as a harmful metabolic byproduct. However, emerging research indicates that lactate has diverse biological functions, encompassing energy regulation, epigenetic remodeling, and signaling activities. Notably, the 2019 study revealed the role of lactate in regulating gene expression through histone and non-histone lactylation, thereby influencing critical biological processes. Metabolic reprogramming is a key adaptive mechanism of cells responding to stresses. The Warburg effect in tumor cells exemplifies this, with glucose preferentially converted to lactate for rapid energy, accompanied by metabolic imbalances, characterized by exacerbated aerobic glycolysis, lactate accumulation, suppressed mitochondrial oxidative phosphorylation, and compromised mitochondrial function, ultimately resulting in a vicious cycle of metabolic dysregulation. As molecular bridges connecting metabolism and epigenetics, lactate and lactylation offer novel therapeutic targets for diseases like cancer and neurodegenerative diseases. This review summarizes the interplay between metabolic reprogramming and mitochondrial dysfunction, while discussing lactate and lactylation’s mechanistic in the pathogenesis of related diseases.

## 1. Introduction

Lactate has long been viewed simply as an end-product of glycolysis and a metabolic waste product. However, there is growing evidence that lactate has a variety of biological functions, including the conversion into pyruvate and production of adenosine triphosphate (ATP) via mitochondrial respiration, synergistic conversion into glucose in the gluconeogenesis pathway, and signal transduction, while also being intricately linked to pathological processes such as the inflammatory response, memory formation, neuroprotection, tissue repair, ischemic injury, and tumorigenesis. In recent years, studies have revealed a new mechanism through which lactate and lactylation regulate gene expression through epigenetic modulation [1,2].

Lactate production is not limited to anaerobic conditions, but also occurs via aerobic glycolysis, which is called the Warburg effect. First observed in tumor cells and later named by Efraim Racker in 1972, the Warburg effect describes aerobic glycolysis in cancer [3]. This metabolic reprogramming enables tumors to secure an energy supply, enhance proliferation, mitigate oxidative stress from oxidative phosphorylation (OXPHOS), and establish an acidic microenvironment to evade immune surveillance [3]. In addition to cancer, aerobic glycolysis is found in sepsis, infections, inflammatory diseases, and autoimmune disorders [4]. Recent research shows that histones are subjected to lysine lactylation in humans and mice [5]. Thus, as a substrate for post-translational modification, lactate regulates gene expression via lactylation at lysine residues on histones and non-histone proteins [6]. Histone lactylation alters the chromatin structure and transcription factor binding, while non-histone lactylation modulates protein stability, activity, and localization [7]. This novel epigenetic modification significantly influences glycolysis [8], macrophage polarization [9], and tumor proliferation [10], underscoring its role in cellular homeostasis and pathogenesis [11].

Metabolic reprogramming, a hallmark of adaptive responses to environmental or pathological stress, includes the Warburg effect in cancer, where glycolysis-derived ATP and lactate persist despite oxygen availability [12]. As the cellular “powerhouses,” mitochondria exhibit functional alterations characterized by either OXPHOS suppression or enhancement during this process [13]. Mitochondrial dysfunction is a common feature of aging and disease, during which it is increasingly correlated with metabolic reprogramming. Under hypoxia, oxidative stress, or nutrient deprivation, cells shift from OXPHOS to glycolysis, leading to lactate accumulation [13]. Crucially, lactate production and mitochondrial dysfunction form a bidirectional “metabolic–mitochondrial–epigenetic” loop, whereby lactate targets mitochondrial complexes, metabolic enzymes, and epigenetic factors, driving disease progression. In tumors, lactate exported via monocarboxylate transporter 1 (MCT1) fosters an immunosuppressive microenvironment while activating oncogenic pathways [14,15]. Neuroendocrine tumors with defective Numb/Parkin signaling display impaired mitophagy, exacerbating glycolytic dependency and lactylation to promote differentiation [16]. In neurodegenerative diseases, lactate acts as a “double-edged sword”, both physiologically supporting axonal regeneration and pathologically inducing mitochondrial dysfunction and oxidative stress via Forkhead box O1/Peroxisome-proliferator-activated receptor gamma coactivator 1 alpha (FOXO1/PGC-1α) [17]. Similarly, acute myocardial infarction triggers a “metabolic collapse–cell death” cascade through H3K18la-mediated apoptosis and an imbalance of mitophagy [18]. Elevated lactate in aging further accelerates tissue degeneration via mitochondrial–lysosomal crosstalk [19].

The current research revolves around the mechanisms of lactate and lactylation in disease, but there are few studies linking it to glycolytic reprogramming and mitochondrial dysfunction. This article reviews the biological roles of lactate and lactylation, with an emphasis on their dual regulatory mechanisms in disease progression mediated through metabolic reprogramming and mitochondrial dysfunction. By integrating recent advances in oncology, as well as the study of neurodegenerative diseases and other disorders, we aim to suggest the intricate link of lactate and lactylation with glycolytic reprogramming and mitochondrial dysfunction. This will deepen our understanding of the link between epigenetics and metabolism, in particular, the ability of lactate and lactylation to synchronize metabolic epigenetic crosstalk, laying the groundwork for the further exploration of their roles in disease development and identification of new therapeutic strategies.

## 2. Mechanisms of Lactate Generation and Lactylation

Glycolysis is recognized as the principal pathway for lactate production [20], but lactate also can be produced via the glutamine pathway. In the classical glycolytic pathway, glucose is activated by hexokinase as it enters the cell and converted by a series of enzymes to produce pyruvate, which is reduced to lactate by lactate dehydrogenase (LDH). Glutamine can be converted through transamination into α-ketoglutarate, which, in turn, enters the tricarboxylic acid cycle, where it can be reduced to pyruvate and, finally, lactate. Glutamine catabolism is mainly used as an alternative source of lactate in cancer cells [21].

The homeostasis of lactate is dependent on a dynamic regulatory network that bridges the gap between glycolytic and aerobic metabolic pathways as both a glycolytic end-product and a substrate for mitochondrial respiration. The regulation of lactate homeostasis can be divided into two pathways, the first being the mitochondrial oxidative pathway, where lactate enters the mitochondria via the monocarboxylate transporter (MCT) and is converted into pyruvate by LDH. The second is the conversion of lactate into glucose via the Cori cycle and the gluconeogenic pathway in hepatocytes and cardiomyocytes. The transcellular transport of lactate is mediated by the MCT protein family, which critically maintains lactate homeostasis through intercellular metabolic coordination. An imbalance of the above mechanisms leads to excessive accumulation of lactate in the serum, ultimately resulting in lactic acidosis, the characteristic metabolic changes of which have been established as prognostic markers for diseases such as sepsis [22,23]. Lactate acts as both a signaling molecule and metabolic substrate, participating in glucose metabolism, fatty acid synthesis, redox homeostasis, and the post-translational modification (PTM) of proteins. It has been demonstrated that lactate can regulate various biological processes such as muscle contraction, wound healing, memory formation, and tumor development [24,25].

As it is a chiral molecule, there are L and D forms of lactate [26]. Mammalian glycolysis predominantly generates L-lactate, including tumor cells subject to the Warburg effect. Under physiological conditions, L-lactate levels significantly exceed those of D-lactate. Therefore, the human body exhibits two distinct protein lactylation pathways, termed direct and indirect lactylation, which arise from the differences in lactate substrates [27]. Histone lysine lactylation (Kla) represents an enzyme-driven process involving three functional components: “writers,” which catalyze the attachment of lactate groups to specific protein sites; “erasers,” responsible for removing lactylation modifications [28], and “readers,” which recognize and interpret lactylation marks through specialized domains. The histone acetyltransferase p300 histone acetyltransferase (p300) directly facilitates lactylation. There is experimental evidence that silencing p300 reduces histone lactylation in the HCT116 human colon cancer cell line (HCT116) and Human Embryonic Kidney 293T (HEK293T) cells, while Sirtuin 1-3 (SIRT1-3) and Histone Deacetylase 1-3 (HDAC1-3) serve as primary lactylation erasers, with HDAC1 and HDAC3 exhibiting robust activity in hydrolyzing both L-lactate and D-lactate modifications [5,26]. The non-enzymatic pathway, first reported by Gaffney et al. in 2020 [29], involves the spontaneous chemical transfer of D-lactate from S-lactoylglutathione to lysine residues. Prevalent in hyperglycolytic tissues, this modification downregulates glycolytic enzymes through feedback inhibition. Intriguingly, L-lactate enhances the stability of S-lactoylglutathione, creating a bidirectional regulatory loop between enzymatic and non-enzymatic lactylation mechanisms [29]. These findings highlight the dual regulatory roles of lactylation in metabolic adaptation and epigenetic modulation. Currently, L-lactate serves as the primary focus in lactylation studies due to its physiological dominance, while the role of D-lactate remains very limited. Thus, the subsequent discussion will center primarily on L-lactate [30].

## 3. Metabolic Reprogramming and Mitochondrial Dysfunction

Metabolic reprogramming represents a cellular adaptation mechanism characterized by the redistribution of metabolic fluxes, typically manifesting as enhanced glycolysis and attenuated oxidative phosphorylation in response to inflammatory or hypoxic stress [31]. As a key biomarker of metabolic reprogramming, the imbalance of lactate homeostasis not only reflects the metabolic state [5], but also triggers a series of biological responses through the accumulation of its metabolic end-products. Mitochondrial dysfunction, a primary driver of this metabolic shift, encompasses reduced biogenesis, impaired energy metabolism, oxidative stress, mitochondrial DNA (mtDNA) abnormalities, and defective mitophagy. Beyond their canonical role as the cellular powerhouse, mitochondria participate in nuclear epigenetic regulation as the organelle that harbors the enzymes of the tricarboxylic acid cycle (TCA) cycle, thereby influencing gene expression patterns. This dual functionality establishes mitochondrial alterations as critical contributors to diseases involving energetic deficits and oxidative damage [32,33].

In rapidly proliferating cells, glycolytic reprogramming maintains cellular homeostasis through two mechanisms, including rapid ATP generation through substrate-level phosphorylation, as well as the maintenance of redox homeostasis through the regulation of the Nicotinamide adenine dinucleotide (Oxidized state)/Nicotinamide adenine dinucleotide (Reduced state) (NAD^+^/NADH) ratio [34]. The lactate produced by this process promotes post-translational protein lactylation. The intricate interplay between glycolytic reprogramming and lactylation exerts significant biological effects, as the increased glycolytic flux elevates lactate production, establishing a direct relationship between the glycolytic flux and protein lactylation levels. This positions lactylation as a crucial nexus connecting metabolic adaptation with mitochondrial function. Lactylation dynamics are bidirectionally regulated by the metabolic balance, as evidenced by the effects of glycolytic inhibitors that deplete lactate and mitochondrial inhibitors or hypoxic conditions that boost lactate generation [5].

**Figure 1:**
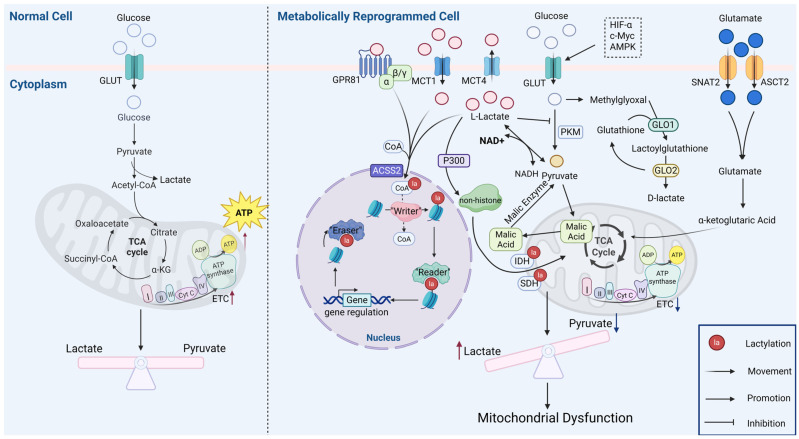
Overview of the interaction between lactate accumulation and mitochondrial dysfunction. (figure was created with Biorender.com).

The above cascade of reactions present is connected to a variety of pathological phenotypes in different diseases. In vascular calcification, lactate orchestrates mitochondrial fission and autophagy via Nuclear Receptor Subfamily 4 Group A Member 1/DNA-Dependent Protein Kinase Catalytic Subunit/Tumor Protein p53 (NR4A1/DNA-PKCs/p53) signaling, activating Dynamin-related protein 1 (Drp1)-mediated fission, disrupting the membrane potential, and impairing respiratory complex activity, which culminate in ATP depletion, reactive oxygen species (ROS) elevation, and the initiation of the mitochondrial apoptotic pathway [35,36,37]. Altered cell membrane permeability is a key trigger of apoptotic signaling [38]. Hypoxic conditions induce the accumulation of mitochondrial alanyl-tRNAsynthetase2 (AARS2), which mediates the lactylation of key OXPHOS enzymes such as pyruvate dehydrogenase E1 subunit alpha 1 (PDHA1) and carnitine palmitoyl transferase 2 (CPT2). This process limits acetyl-CoA availability, thereby suppressing OXPHOS and, ultimately, impairing mitochondrial functional integrity [39]. In pulmonary arterial hypertension, hypoxic mitochondrial reactive oxygen species (mtROS) inhibit the hydroxylation of hypoxia inducible factor-1α (HIF-1α), activating the HIF-1α/PDK1-2/p-PDH-E1α axis (hypoxia inducible factor-1α/Pyruvate dehydrogenase kinase isozyme1-2/phosphorylated pyruvate dehydrogenase E1 alpha subunit, HIF-1α/PDK1-2/p-PDH-E1α) to enhance glycolysis, lactate accumulation, and histone lactylation (H3K18/H4K5la), driving the proliferation of smooth muscle cells in pulmonary artery walls [40]. Systemic lupus erythematosus patients exhibit mtDNA-release-induced metabolic reprogramming, characterized by the glycolytic upregulation of monocytes and macrophages, mitochondrial impairment, and lactate-driven cyclic guanosine monophosphate–adenosine monophosphate synthase (cGAS) lactylation that activates cyclic guanosine monophosphate–adenosine monophosphate synthase stimulator of interferon genes—Interferon alpha 1 (cGAS-STING-IFN1) signaling that aggravates inflammation [41,42,43]. Myocardial ischemia–reperfusion injury features OXPHOS suppression with compensatory glycolysis, whereby the K241 lactylation of malate dehydrogenase 2 (MDH2), a key enzyme of the TCA cycle, promotes ferroptosis and mitochondrial dysfunction [44]. Collectively, these findings suggest that lactate and post-translational protein lactylation play a dual role in metabolic adaptation and mitochondrial damage [45]. A schematic illustrating the relationship between metabolic changes and mitochondrial function is shown in Figure 1. Table 1 shows lactylation targets that have an important role in mitochondrial function.

In normal cells, glucose enters the cell via glucose transporters and undergoes glycolysis to generate pyruvate, which is then transported into mitochondria to produce substantial energy for cellular functions. In metabolically reprogrammed cells, glucose also enters the cell through glucose transporters and is converted into L-lactate via glycolysis. In this process, factors such as HIFα, c-Myc, and AMPK promote lactate production by enhancing the expression of glycolytic enzymes and glucose transporters. Cells can indirectly generate D-lactate through the glutaminolysis pathway. Lactate enters the nucleus to modify histones under the action of ACSS2 enzyme, constituting a process of direct lactylation, which enters the nucleus to modify histones, thereby regulating gene expression and inducing the corresponding cellular phenotypic changes, while another portion of lactate leads to the lactylation modification of non-histone proteins under the action of P300, which impairs the mitochondrial function through the modification of the TCA cycle enzymes, exacerbating metabolic reprogramming and mitochondrial inhibition. (GLUT: Glucose Transporter, MCT4: Monocarboxylate transporter 4, GPR81: G protein—coupled receptor 81, SNAT2: Sodium coupled neutral amino acid transporter 2, ASCT2: Alanine serine cysteine transporter, ADP: Adenosine diphosphate, AMPK: Adenosine 5′-monophosphate-activated protein kinase, ACSS2: Acetyl-CoA synthetase 2, GLO1: Glyoxalase 1, GLO2: Glyoxalase 2, SDH: Succinate dehydrogenase, IDH: Isocitrate dehydrogenase, ETC: Electron transport chain).

## 4. Cancer

Lactate and lactylation serve as critical mediators bridging glycolytic reprogramming with OXPHOS dysfunction in cancer. In general, tumors are characterized by the dysregulation of epigenetic modifications and metabolic activities [53]. Mitochondrial dysfunction drives tumor metabolic reprogramming, with neoplastic mitochondria showing structural and functional defects and a heterogeneous quantity and morphology compared to normal counterparts [54,55]. At the same time, a bioenergetic crisis emerges during this process. To address this, tumor cells activate metabolic reprogramming by initiating aerobic glycolysis, a key component of the Warburg effect [56]. For example, the dysfunction of the mitochondrial ETC decreases the electron transfer efficiency and OXPHOS capacity, leading to impaired ETC complex activity, reduced ATP synthesis, and compromised OXPHOS output [57]. Key manifestations include impaired NAD^+^ regeneration and an imbalanced NAD^+^/NADH ratio, which drive pyruvate conversion into lactate and increased glycolytic flux [58]. Additionally, the inactivation of the mitochondrial pyruvate dehydrogenase complex (PDC) drives the metabolic shift from OXPHOS toward aerobic glycolysis in cancer cells, which is primarily attributed to reduced activity of TCA-cycle-associated enzymes. As PDC is a central hub linking glycolysis with the TCA cycle, its dysfunction results in intracellular lactate accumulation. The resulting blockade of pyruvate entry into the TCA cycle further impairs OXPHOS and promotes histone lactylation (H3K56la) [46]. Mechanistically, p300 acetylates the mitochondrial protein pyruvate dehydrogenase complex component X (PDHX) at the cytoplasmic K488 site, thereby destabilizing PDC assembly and enhancing glycolytic flux. The resulting lactate surge promotes H3K56la-mediated chromatin remodeling and pro-tumorigenic gene expression [59,60].

Glycolytic reprogramming is a key metabolic adaptation in tumor cells, driving development through two main mechanisms: supplying energy for rapid proliferation and enabling adaptation to hypoxia, thereby promoting survival and metastasis [56]. Furthermore, glycolysis generates substantial amounts of lactate, which is transported via monocarboxylate transporters (MCTs) in tumor cells, identifying the tumor microenvironment (TME), impairing immune cell function, disrupting intracellular lactylation levels, and, ultimately, destabilizing the transcriptional equilibrium to drive tumorigenesis [49]. Lactate also functions as a signaling molecule in tumor progression. For example, lactate secreted into the TME by tumor cells acts on G-protein-coupled receptor 81 (GPR81) expressed on malignant cells, adjacent stromal components, and non-cancerous cells (T cells and macrophages within the TME, promoting tumor growth and metastasis. It has been reported that phosphoinositide 3-kinase/protein kinase B (PI3K/Akt) pathway activation can suppress tumor development [61]. Moreover, the inhibition of GPR81 signaling suppressed the pro-angiogenic mediator amphiregulin (AREG) via PI3K/Akt/cAMP-dependent pathways. GPR81 knockdown significantly inhibited cancer cell proliferation and metastasis, accompanied by markedly reduced mitochondrial activity and increased apoptosis [62].

Disease-specific manifestations reveal the complexity of these mechanisms. Lactate not only acts as a signaling molecule but also serves as a substrate for post-translational modifications on histone lysine residues, disrupting transcriptional homeostasis and promoting oncogenesis [63]. Specifically, histone lactylation (H3K56la) activates oncogenic drivers such as *Myelocytomatosis Oncogene* (*MYC*) and *Cyclin D1 gene* (*CCND1*), forming a synergistic metabolic–epigenetic network that drives malignant transformation [64]. Tumor tissues exhibit elevated histone lactylation levels compared to normal tissues, and higher levels are correlated with a poor patient prognosis. In clear cell renal cell carcinoma, histone lactylation promotes a Platelet Derived Growth Factor Receptor Beta (PDGFR-β)-mediated positive feedback loop that drives aggressive disease progression [65]. The N6-Methyladenosine (m6A) modification plays critical roles in cancer progression [66], while histone lactylation enhances m6A site binding, leading to *period1* Circadian Regulator 1 (*PER1*) and *Cellular tumor antigen p53* (*TP53*) degradation ocular melanoma [10]. In gliomas, H3K18la activates *methyltransferase like protein 3* (*METTL3*) transcription and mediates RNA m6A modifications to foster immunosuppression in tumor-infiltrating myeloid cells. H3K9la is strongly enriched in the *LUC7 like 2*, *pre-mRNA splicing factor* (*LUC7L2*) promoter region, stimulating its transcription [67]. Histone lactylation drives *METTL3*-mediated RNA m6A modifications, which are pivotal in enhancing the immunosuppressive capacity of tumor-infiltrating myeloid cells (TIMs).

Beyond histone modifications, the lactylation of non-histone proteins also significantly promotes disease progression. In prostate cancer, lactate stabilizes HIF-α via lactylation under normoxic conditions, regulating downstream pathways and highlighting the diverse mechanisms of lactylation in tumorigenesis [68]. In hepatocellular carcinoma (HCC), the Kla protein predominantly affects enzymes involved in the TCA cycle and fatty acid metabolism. Kla modification at site 28 promotes HCC proliferation and metastasis by inhibiting adenylate kinase 2 (AK2). Hypoxic HCC models exhibited an upregulation of glypican-3 (GPC3), which elevates c-Myc lactylation levels, enhancing its stability and expression to drive HCC progression [69]. Recently, molecular-targeted therapy for hepatocellular carcinoma has expanded from single-kinase inhibitors to multidimensional intervention strategies, covering targets such as anti-angiogenesis and immune microenvironment remodeling, as well as combination therapy and biomarker-guided precision typing, which are driving breakthroughs in clinical efficacy [70]. Linking metabolism and epigenetics will inform the discovery of new molecularly targeted therapies for liver cancer.

**Figure 2:**
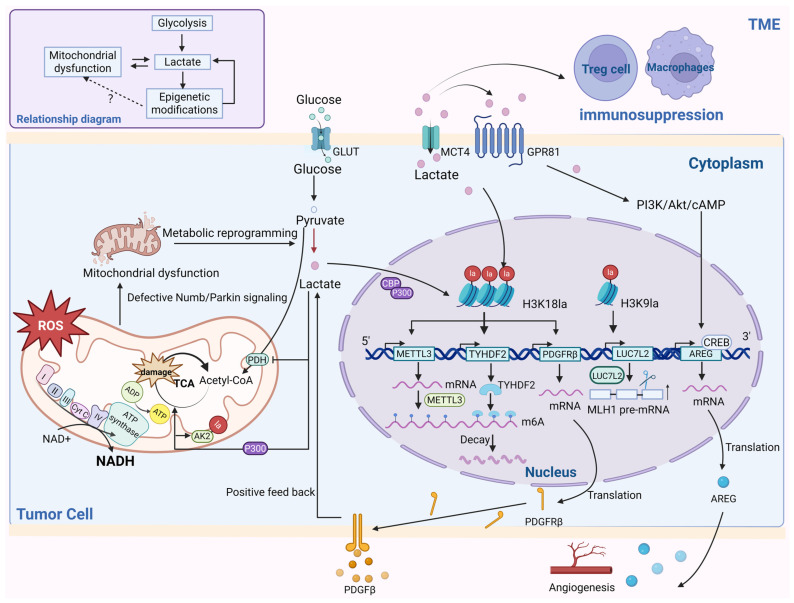
The role of lactate accumulation and lactylation in tumors. (figure was created with Biorender.com).

Lactate and lactylation are pivotal regulators of metabolism and mitochondrial function, promoting pathogenesis through epigenetic modulation. In non-small-cell lung cancer cells, lactate attenuates glucose uptake and glycolysis to maintain mitochondrial homeostasis. Glycolytic enzymes and TCA cycle enzymes exhibit downregulated and upregulated mRNA levels, respectively, with increased histone lactylation at the *Hexokinase 1* (*HK-1*) and *Isocitrate Dehydrogenase* (*NAD*(+) *3 Non-Catalytic* (*IDH3G*) promoters [71]. Lactate regulates cellular metabolism via histone-lactylation-mediated gene expression. Studies have identified fragmented mitochondria with a low membrane potential in neuroendocrine prostate or lung cancer cells, where Numb interacts with Parkin to promote Parkin-mediated mitophagy. Defects in the Numb/Parkin pathway in prostate or lung adenocarcinoma drive metabolic reprogramming, forcing cells to rely on aerobic glycolysis for energy production and survival under mitochondrial dysfunction. Concurrent lactate overproduction induces Pan-Kla and histone lactylation (H3K18). Pan-Kla signals activate transcription start site (TSS)-proximal regions of *MYCN*; this is a key transcription factor that promotes the neuroendocrine differentiation of PCa, and histone lactylation can activate the significant enrichment of neuroendocrine genes, such as *Neural Cell Adhesion Molecule 1* (*NCAM1*) and *Enolase 2* (*ENO2*). Numb/Parkin-directed mitochondrial adaptation acts as a critical metabolic switch, in which mitochondrial dysfunction triggers metabolic reprogramming and lactate accumulation. These processes not only alter tumor cell energy metabolism and histone lactylation levels, but also promote neuroendocrine differentiation via TME modulation [16]. Elevated histone lactylation increases neuroendocrine gene expression, revealing novel mechanisms linking tumor metabolism with epigenetic regulation, potentially leading to new therapeutic targets. The mechanisms underlying the roles of lactate and lactylation in tumors are illustrated in Figure 2. These findings show the intricate interplay between tumor cell metabolism and epigenetic regulation, providing innovative strategies for targeted cancer therapy.

Tumor cell mitochondrial abnormalities are manifested by mitochondrial electron transport chain ETC dysfunction, resulting in impaired NAD^+^ regeneration and an imbalance of the NAD^+^\NADH ratio, as well as mitochondrial autophagy disorders, etc. The cell responds to the energy crisis by activating metabolic reprogramming through the initiation of aerobic glycolysis, and the lactate produced during this process, in turn, affects the activity of related enzymes in the mitochondria, exacerbating the mitochondrial dysfunction and increasing glycolytic flux, and the resulting lactate enters the nucleus to promote the expression of relevant genes through epigenetic modifications, such as *METTL3*, *TYHDF2*, *PDGFRB*, *LUC7L2*, etc., which further contribute to the development of the disease, or lactate affects the development of the disease in a way that activates the signaling pathway. Lactate can also be secreted into the tumor microenvironment via MCT4 causing immunosuppression. The interactions between tumor metabolism and epigenetics are intricate, and there is relatively little evidence to suggest a link between epigenetic regulation and mitochondrial dysfunction. (P300\CBP: CREB-Binding protein, P300: E1A-binding protein).

## 5. Neurological Disorders

As a metabolic byproduct of glycolysis, lactate also serves as an energy substrate for neurons, facilitating the protection and regeneration of damaged axons. It holds significant implications for neurological disorders, which are typically characterized by complex pathological processes including neural cell damage and inflammation. However, the chronic excessive accumulation of lactate disrupts intracellular acid–base homeostasis, leading to an acidotic state. This acidic environment impairs mitochondrial function, leading to the production of ROS, thereby exacerbating oxidative stress [72]. Moreover, lactate can also modulate various intracellular signaling pathways, including those involved in inflammation and neurodegenerative pathologies, leading to overall neuronal damage. The concept of glycolytic reprogramming, originally described as the Warburg effect in cancer, manifests in neural systems through dynamic shifts between oxidative phosphorylation and aerobic glycolysis [73]. This metabolic shift also exists in the nervous system, where ATP required by neurons is produced from glucose via OXPHOS in the mitochondria [74]. Dysregulation at any node of neuronal glucose metabolism compromises motor and cognitive functions, including learning and memory consolidation. Mechanistically, lactate operates as both the metabolic fuel and signaling mediator [75].

Lactate produced by glucose metabolism in neuronal cells has an important impact on the course of neurological diseases. Some studies have shown that, in hypothalamic pro-opiomelanocortin (POMC) neurons, lactate modulates feeding behavior and peripheral glucose metabolism via mitochondrial uncoupling protein 2 (UCP2) dependent redox signaling [76]. Hyperglycemia triggers metabolic inflexibility in neurons, shifting energy production from OXPHOS to glycolysis. This pathological rewiring induces mitochondrial dysfunction and lactate overproduction, particularly in microglia, where the accumulated lactate drives Pan-Kla modifications. Studies have shown that the level of pan-histone lysine lactylation is significantly increased in senescent microglia of naturally senescent mice and AD mice; histone lactylation (H3K18), especially, can stimulate the nuclear factor kappa-B (NF-κB) signaling pathway, thereby upregulating the aging-related secretory phenotypic components IL-6 and IL-8 [77]. Another study showed that histone lactylation (H3K9la) in microglia could activate the expression of *Solute Carrier Family 7 Member* (*SLC7A*) and promote microglial activation [78]. Histone lactylation (H4K12) exacerbates microglial dysfunction [47]. H4K12 lactylation stimulates FOXO1/PGC-1α signaling, ultimately exacerbating mitochondrial oxidative stress and diabetic cognitive impairment [17]. As the precursor for Kla, lactate critically regulates this post-translational modification [79]. Investigating the role of Kla in central nervous system (CNS) disorders will not only elucidate fundamental neurophysiological mechanisms, but also open therapeutic avenues targeting metabolic–epigenetic crosstalk.

### 5.1. Ischemia–Reperfusion Injury

**Figure 3:**
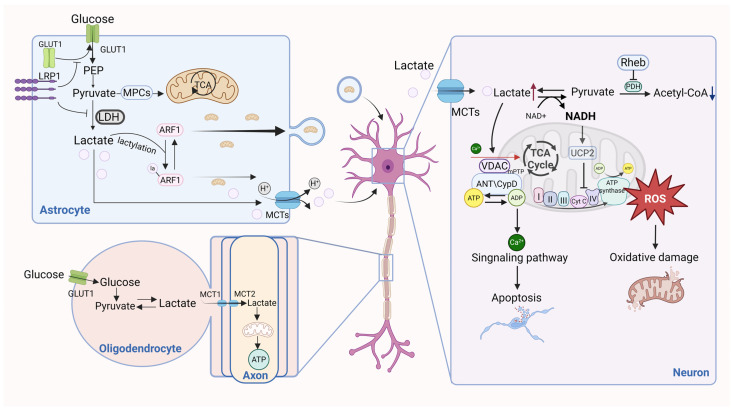
The role of lactate and lactylation in nerve cells. (figure was created with Biorender.com).

Ischemia–reperfusion injury occurs when blood supply is suddenly restored to an organ after a period of ischemia, resulting in a large Ca^2+^ influx and the generation of a large amount of oxygen free radicals after reperfusion, which is the main pathogenic mechanism of widespread tissue injury [80]. Ischemia-induced metabolic acidosis is an important cause of cellular dysfunction and metabolic disturbances, but the rapid correction of acidosis in ischemic tissues during reperfusion actually exacerbates cellular injury [81]. Studies have confirmed that acidosis in ischemic regions exacerbates metabolic failure by depleting the intracellular energy supply, causing acidosis due to massive lactate accumulation and, consequently, cellular metabolic dysregulation, thereby disrupting protein synthesis, while inducing neuronal excitotoxicity through the dysregulation of sodium channel kinetics [50]. Voltage-dependent anion channel 1 (VDAC1), the most abundant mitochondrial outer membrane porin, facilitates calcium influx and small-molecule transport across mitochondrial membranes [50]. Notably, there is a close relationship between cytoplasmic calcium concentration and mitochondrial function [82]. Mass spectrometry analyses in rat models of cerebral ischemia–reperfusion injury (CIRI) identified two lactylated adenine nucleotide translocator (ANT) isoforms, whereby VDAC1 lactylation was also detected, which suggests a critical role for lactylation in calcium-overload-associated CIRI. However, reduced VDAC1 lactylation in CIRI rat brain endothelia impaired mitochondrial apoptotic pathways, accelerating neuronal death [50]. Astrocytes, pivotal for cerebral homeostasis and neuroprotection, transfer mitochondria to damaged neurons [83]. Mitochondrial dynamics play an important role in numerous diseases [84]. Notably, low-density lipoprotein receptor-related protein 1 (LRP1) influences this process, as LRP1 knockdown suppressed the transfer of mitochondria from astrocytes, diminished neuroprotection, and enhanced glycolysis with concurrent lactate accumulation. The K73 delactylation of ARF1 enhances mitochondrial export, while persistent lactylation inhibits transport [48]. LRP1 mitigates astrocytic glycolysis and lactate production, thereby reducing ARF1K73 lactylation to promote mitochondrial transfer to injured neurons, revealing a novel lactylation-dependent neuroprotective mechanism and therapeutic target for ischemic stroke. The role of lactate and lactylation in neuronal cells is illustrated in Figure 3.

The excessive accumulation of lactate in neuronal cells produces an acidic environment that impairs mitochondrial function and enhances reactive oxygen species production, exacerbating oxidative stress, and also mediates calcium inward flow and mitochondrial apoptotic pathways via voltage-dependent anion channel protein lactylation. Astrocytes can support energy metabolism by shuttling lactate into neurons through the extracellular fluid, and, in addition, LRP1 enhances mitochondrial translocation to support damaged neurons by inhibiting glycolysis and lactate production in astrocytes to promote the delactylation of ARF1. Oligodendrocytes rely on glycolysis to generate energy and form electrical insulation around axons, resulting in facilitating the electrical conduction of action potentials while providing metabolic and nutritional support to axons. (GLUT1: Glucose transporter 1, LRP1: Low density lipoprotein receptor-related protein 1, PEP: Phosphoenolpyruvate, PDH: Pyruvate dehydrogenase, MCT2: Monocarboxylate transporters 2, Rheb: Ras homolog enriched in brain, CypD: Cyclophilin D, MPCS: Membrane protein complexes, UCP2: Uncoupling protein 2).

### 5.2. Neurodegenerative Diseases

Energy homeostasis serves as the fundamental basis for maintaining normal physiological functions in brain tissues, while metabolic dysregulation is a primary driver of neurodegenerative disorders, including Alzheimer’s (AD) and Parkinson’s disease (PD) [85]. These disorders manifest distinct pathological alterations, including oxidative stress, mitochondrial dysfunction, and autophagic impairment, which depend on the specific affected neuronal populations and their anatomical localization [86]. Within the neuron–glia metabolic coupling system of the brain, glycolytic astrocytes export lactate as an energy substrate for adjacent neurons. Notably, astrocytes continue to generate substantial amounts of lactate via aerobic glycolysis even under oxygen-replete conditions [87]. Research demonstrates that astrocyte-derived lactate can diffuse through the extracellular fluid into neurons [88]. Both astrocytes and neurons express MCTs that co-transport lactate and hydrogen ions. Lactate released from astrocytes is transferred into neuronal mitochondria, where it serves as a TCA cycle substrate [75], providing energy to neurons. This lactate-derived energy supply meets the metabolic needs of neurons, directly supporting synaptic transmission and neural activity while modulating glycolytic inhibition and metabolic regulation. The experimental blockade of lactate transfer from astrocytes to neurons induces neuronal lactate deficiency, leading to energy depletion that triggers mitochondrial dysfunction and oxidative stress. These pathological cascades ultimately exacerbate neuronal damage [89]. Oligodendrocytes are myelinating cells within the CNS that wrap around axons to form electrically insulating structures, facilitating the efficient propagation of neuronal action potentials while providing metabolic and trophic support to axons. Similar to astrocytes, mature oligodendrocytes exhibit a strong reliance on glycolysis for energy production even under oxygen-replete conditions [90]. Furthermore, glutamate released by neurons binds to glutamate receptors on glial cells, which modulate the transport of lactate from both oligodendrocytes and astrocytes to neurons [91].

AD is intrinsically linked to neuronal metabolic states, with pathogenesis driven by aberrant microglial activation and senescence. Microglia, responsible for phagocytosing neurotoxic debris and releasing cytotoxic factors, initially protect the CNS through antigen presentation and the secretion of pro-inflammatory cytokines [75,77,92]. Activated microglia secrete pro-inflammatory cytokines and chemokines to protect the central nervous system from further damage [47]. However, during this process, microglia undergo metabolic reprogramming from mitochondrial oxidative phosphorylation to aerobic glycolysis in response to pathological stimuli [93]. This persistent stress induces aerobic glycolysis, which drives excessive lactate production and stimulates the production of inflammatory mediators, disrupts mitochondrial metabolism, and impairs microglial phagocytic as well as chemotactic functions. Paradoxically, these alterations exacerbate neurotoxicity while promoting self-perpetuating cycles where damaged neurons trigger the activation of neighboring microglia, ultimately accelerating amyloid-β plaque deposition [94,95]. Mechanistically, glycolytic reprogramming elevates the levels of lactate, which serves as a critical mediator enhancing the release of pro-inflammatory factors from microglia.

Increased lactate promotes histone lactylation and regulates the expression of specific genes affecting disease processes. In AD patients, microglia surrounding amyloid plaques demonstrate elevated histone lactylation (H4K12la), which upregulates the expression of pyruvate kinase M2 (PKM2). This creates a self-reinforcing loop: PKM2-driven glycolysis amplifies lactate synthesis, further increasing H4K12la levels, thereby intensifying microglial inflammatory activation and neuroinflammation to drive disease progression [47]. Histone lactylation modulates specific gene networks in microglia, ultimately inducing neuronal damage and cognitive decline. The experimental attenuation of microglial glycolysis and histone lactylation ameliorated Aβ pathology and cognitive deficits [47]. Notably, Aβ deposition also disrupts aerobic glycolysis in astrocytes [96]. As primary glycolytic hubs, astrocytes convert glucose into lactate for neuronal uptake and mitochondrial pyruvate metabolism [97]. Remarkably, glucose hypometabolism and mitochondrial dysfunction may be early biomarkers preceding Aβ accumulation [98].

Additional studies reveal age-dependent and AD-associated increases in histone lactylation (H3K18la) within glial cells and hippocampal neurons, modulating the NF-κB signaling pathway to attenuate neuroinflammation [99]. H3K18la activates NF-κB signaling to generate senescence-associated secretory phenotypes (SASPs), exacerbating brain aging and AD pathophysiology [77]. Spatially distinct distributions of H4K12la and H3K18la in AD brains play differential biological roles, suggesting that therapeutic strategies targeting specific lactylation sites may intercept disease progression. As neuronal powerhouses, mitochondria regulate energy homeostasis through ATP synthesis and ROS generation. While physiological ROS levels are byproducts of aerobic metabolism, respiratory chain dysfunction causes ROS overproduction, which is a key mechanism linking mitochondrial impairment to AD pathogenesis [100]. Clinically, elevated lactate levels in the cerebrospinal fluid of AD patients reflect a mitochondrial deficiency in the central nervous system [101]. Therapeutic interventions using glucagon-like peptide-1 (GLP-1) receptor agonists demonstrate dual benefits, enhancing the expression of glycolytic enzymes to reduce the burden of oxidative phosphorylation, thereby lowering ROS production while increasing ATP synthesis to meet neuronal energy demands [102]. Elevating intracellular NADH levels promote lactate production while restricting pyruvate entry into mitochondria to preserve mitochondrial integrity, thereby alleviating oxidative stress and ameliorating AD symptoms [103]. Overall, attenuating oxidative damage and restoring mitochondrial function may be promising therapeutic strategies for the management of AD.

During AD progression, neurons undergo metabolic reprogramming from oxidative phosphorylation to glycolysis. This cell-type-specific metabolic shift together with mitochondrial dysfunction disrupts the metabolic coupling between neuronal cells and drives AD pathology. Studies have shown that the inhibition of PKM2 can block the positive feedback loop of glycolysis–H4K12la–PKM2, inhibit the expression of glycolysis-related genes, reverse metabolic reprogramming, and reduce lactate levels, thereby alleviating microglia activation and dysfunction [47]. Current research on the relationship between glucose metabolic reprogramming and mitochondrial dysfunction remains incomplete, with evidence primarily derived from in vitro experiments. Elucidating the causal relationships underlying neural-cell-specific and brain-region-dependent alterations of glucose metabolism is critical for deciphering disease mechanisms.

PD is the second most common neurodegenerative disorder. Its primary pathological mechanism involves the degeneration of dopaminergic neurons projecting from the substantia nigra pars compacta to the striatum, leading to impaired dopamine neurotransmission and core motor symptoms such as resting tremor, bradykinesia, rigidity, and postural instability [104]. Oxidative stress, lysosomal dysfunction, and mitochondrial impairment are closely associated with the pathophysiology of PD [105].

**Figure 4:**
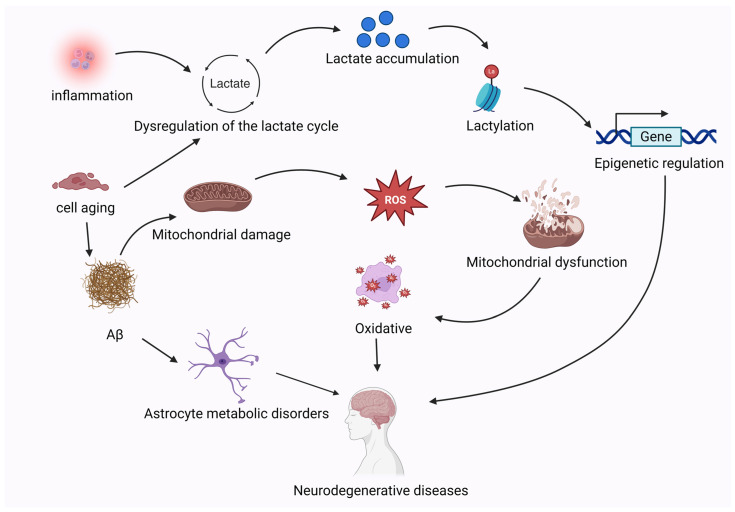
A vicious cycle triggered by an imbalance in lactate metabolism in neurodegenerative diseases. (figure was created with Biorender.com).

It has been shown that the upregulation of HK2 and LDHA, as well as elevated levels of lactate, promote apoptosis in PD, while the inhibition of HK2 expression attenuates the apoptosis of dopaminergic neurons through decreased lactate production and the downregulation of the AMP-activated protein kinase/Protein kinase B/mammalian target of rapamycin (AMPK/Akt/mTOR) pathway in PD [106]. Another study indicated that AMPK activation exerts neuroprotective effects by restoring mitochondrial function and suppressing glycolysis-dependent metabolic shifts, thereby reducing lactate accumulation. Conversely, AMPK inhibition reduces the mitochondrial DNA content and energy supply while decreasing astrocyte survival, indirectly exacerbating neuronal apoptosis [106,107,108]. Notably, under high-glucose conditions, AMPK activation irreversibly inhibits astrocyte proliferation, enhances glycolysis, and elevates both lactate and ATP levels, suggesting a potential link between AMPK signaling and metabolic reprogramming [107]. Furthermore, AMPK activates the PINK1/Parkin pathway to ameliorate mitochondrial dysfunction in PD models [109]. The dysregulation of lactate metabolism is emerging as a key mechanism in PD. Excessive lactate production may serve as an indirect biomarker of mitochondrial dysfunction, while lactate accumulation may influence disease progression through protein lactylation. Intriguingly, the 14-3-3 protein family suppresses inflammatory responses in cerebral tissues through lactylation-dependent binding to the pyrin domain of NOD-like receptor thermal protein domain associated protein 3 (NLRP3), thereby inhibiting NLRP3 inflammasome activation and the subsequent mitochondrial pyroptosis mediated by gasdermin D (GSDMD)-NT fragments [110,111]. This process prevents mitochondrial DNA leakage and mitigates neuroinflammation, highlighting the dual role of lactate as both a metabolic marker and a regulator of inflammatory pathways [112]. The vicious circle triggered by the metabolic imbalance in neurodegenerative diseases is illustrated in Figure 4.

The pathophysiology of PD is closely associated with oxidative stress, mitochondrial dysfunction, and lysosomal impairment. AMPK activation exerts neuroprotective effects by restoring mitochondrial function, suppressing glycolysis-dependent metabolic shifts, and reducing lactate accumulation. However, excessive AMPK inhibition has been shown to result in mitochondrial DNA depletion, energy insufficiency, and reduced astrocyte survival, indirectly exacerbating neuronal apoptosis. Lactate not only serves as a biomarker of mitochondrial dysfunction, but is also emerging as a novel therapeutic target for modulating inflammatory and cell death pathways. PD research has expanded beyond the sole focus on neuronal protection to exploring multidimensional metabolic–immune–mitochondrial regulatory networks, paving the way for innovative therapies targeting metabolic reprogramming and the modulation of inflammatory pathways.

Cellular senescence causes the production of amyloid plaques in nerve cells, and the metabolic reprogramming of oxidative phosphorylation to glycolysis, affecting the function of astrocytes; the metabolic homeostasis of cells is broken, and lactate cannot be removed normally, and the abnormal accumulation of lactate directly damages mitochondria, which reduces ATP production, releases a large amount of reactive oxygen species, triggers oxidative stress, and further destroys the cell structure and causes diseases. (Aβ: amyloid-β).

## 6. Other Diseases

Lactate and lactylation play key roles in the development of various diseases, forming critical bridges between metabolic reprogramming and epigenetic regulation.

During myocardial infarction, the sudden onset of hypoxia enforces anaerobic metabolism in cardiomyocytes, resulting in pathological lactate accumulation. This metabolic shift promotes histone lysine lactylation, which regulates gene expression. In addition to glucose deficiency, the energy crisis is exacerbated by mitochondrial dysfunction due to the inability of mitochondria to synthesize ATP through OXPHOS, which triggers cytochrome C release, activates caspase-9, and, ultimately, induces apoptosome formation and cardiomyocyte death [18,38,113]. In cardiac tissues of acute myocardial infarction (AMI) patients, mitochondrial fragmentation, diminished membrane potential, and disrupted mitophagic homeostasis are observed. Notably, lactylation forms part of a positive feedback mechanism, regulating gene networks to partially restore mitochondrial metabolic equilibrium. Mitochondrial dynamics, particularly fusion–fission balance, critically influence macrophage functionality during inflammatory responses. Excessive mitochondrial fission elevates intracellular lactate levels, upregulates arginase-1 expression, promotes M2 macrophage polarization, enhances the phagocytic capacity, and mitigates inflammatory damage [114]. Lactate-dehydrogenase-mediated metabolic reprogramming alleviates ROS accumulation due to mitochondrial dysfunction, while inducing M2 polarization, collectively promoting cardiomyocyte proliferation. Disordered glycolysis and dysregulated MCT1-mediated lactate transport induce histone lactylation (H3K18), which modulates the anti-inflammatory responses of monocytes and macrophages to prevent the functional deterioration of cardiac muscle [114].

Cellular senescence is characterized by irreversible growth arrest, which arises from mitochondrial dysfunction and epigenetic alterations [19]. Metabolic derangement and mitochondrial impairment force cells to shift from oxidative phosphorylation to glycolysis, generating excessive lactate that exacerbates metabolic dysfunction [115]. TNF receptor-associated protein 1 (TRAP1), a senescence-associated mitochondrial gene regulating cellular metabolism and lactate homeostasis, accelerates senescence in vascular smooth muscle cells (VSMCs). VSMC senescence involves metabolic reprogramming with lactate accumulation, whereby TRAP1 deficiency improves mitochondrial function while its overexpression accelerates senescence. VSMC-specific TRAP1 knockout attenuated senescence and atherosclerosis via metabolic reprogramming. TRAP1 overexpression enhanced aerobic glycolysis and lactate production, which, in turn, downregulated histone deacetylase HDAC3 to promote histone lactylation (H4K12la). H4K12la enrichment at SASP promoters activates SASP transcription, aggravating the aging of smooth muscle [116,117]. The crosstalk between mitochondria and the nucleus is important for maintaining cellular homeostasis, whereby the energy metabolism of the cell provides specific substrates or cofactors for histone modifications that regulate gene transcription and contribute to disease development.

In sepsis, pyruvate dehydrogenase alpha 1 (PDHA1) is predominantly upregulated in renal tubular epithelial cells, where its acetylation-mediated inactivation drives excessive lactate production. This lactate surge mechanistically induces the lysine 20 lactylation (K20la) of mitochondrial fission 1 protein (Fis1). Elevated Fis1 K20la promotes pathological mitochondrial hyper-fission, triggering ATP depletion, mitochondrial ROS overproduction, and apoptotic signaling cascades, ultimately exacerbating sepsis-associated acute kidney injury (SAKI) [51]. There is experimental evidence that *METTL15* deficiency induces mitochondrial dysfunction characterized by increased ROS, diminished membrane potential, and altered metabolic states. Crucially, *METTL15* knockout models demonstrate the concomitant elevation of lactate secretion and histone lactylation levels (H4K12la and H3K91la), positioning *METTL15* as a promising regulatory node for investigating the crosstalk between mitochondrial metabolism and histone lactylation reprogramming during sepsis [118].

Mitochondrial dysfunction is a central mechanism of drug-induced liver injury (DILI), often accompanied by elevated lactate levels. Acetaminophen (APAP) treatment suppresses the SIRT1/PGC-1α/LDHB axis, which regulates mitochondrial quality control and lactate metabolism. This suppression increases mitochondrial protein lactylation and intramitochondrial lactate levels in APAP-induced hepatotoxicity. The overexpression of peroxisome-proliferator-activated receptor γ coactivator 1 alpha (PGC-1α) or the administration of its activators ameliorates APAP-induced damage in hepatocytes and murine liver tissues, enhances lactate dehydrogenase B (LDHB) synthesis, and reduces mitochondrial protein lactylation and lactate accumulation [119].

In acute kidney injury (AKI), lactylation has been demonstrated to exacerbate mitochondrial dysfunction. The lactylation of aldehyde dehydrogenase 2 (ALDH2) at lysine 52 (K52la) exacerbates tubular injury and mitochondrial dysfunction. ALDH2 lactylation promotes the ubiquitination-induced proteasomal degradation of prohibitin2 (PHB2) to inhibit mitophagy and worsen mitochondrial dysfunction [52,120]. The mechanisms related to other diseases are shown in Table 2.

As an end-product of glycolysis, lactate accumulates in myocardial infarction, cellular senescence, sepsis, and drug-induced liver injury through metabolic reprogramming, which drives the lactylation of histones and mitochondrial proteins, thus forming a chain from metabolic signaling to epigenetic regulation, while impaired oxidative phosphorylation in mitochondria triggers an increase of compensatory glycolysis, resulting in an amplification of pathological cascade effects. Therefore, targeting the lactate metabolic pathway and related epigenetic modifications may become a new strategy to intervene in disease progression [121].

**Table 2:** Molecular mechanisms of diseases related to regulation of mitochondrial function by lactate.

Category	Mechanism	Detail	Reference
APAP-induced liver injury	Inactivation of SIRT1\PGC-1α\LDHB	Decreasing SIRT1/PGC-1α/LDHB expression leads to metabolism reprogramming, and increased protein lactylation, mitochondrial lactate levels, and pathological damage in liver mitochondria. PGC-1α overexpression increased LDHB synthesis, reduced lactylation, and induced a switch from lactate to pyruvate production.	[119]
Hepatic ferroptosis	Inactivation of Parkin\OXSM	Lactate activates mitochondrial phosphoenolpyruvate carboxykinase 2 (PCK2) through KAT8-mediated lactylation modification. This activation suppresses Parkin-mediated ubiquitination degradation of 3-oxoacyl-ACP synthase (OXSM), leading to metabolic reprogramming of mitochondrial fatty acid synthesis (mtFAS).	[122]
Sepsis induced acute kidney injury	Activation of Fis 1	Pathological stimulation leads to metabolism reprogramming, lactate accumulation mediates lysine 20 lactylation (K20la) of mitochondrial fission 1 protein (Fis1), and elevated Fis1 K20la promotes excessive mitochondrial fission, resulting in ATP depletion, overproduction of mitochondrial reactive oxygen species (ROS), and mitochondrial apoptosis.	[51]
Pulmonary fibrosis	Inactivation of ERK/DRP1	Lactate produced by metabolic reprogramming could promote lung fibrosis by increasing mitochondrial fission-derived ROS via ERK/DRP1 signaling.	[123]
Inflammatory responses in macrophages	Overexpression of Arg 1	Mitochondrial-fragmentation-caused metabolic reprogramming leads to increase pan-histone lactylation, which caused an increase in arginase 1 expression, which promotes Inflammation Resolution Responses.	[114]
Vascular calcification	Dysfunction of PARP1\POLG\UCP2	Lactate induced the translocation of PARP1 from the nucleus to mitochondria, where it subsequently bound to DNA polymerase gamma catalytic subunit (POLG) and inhibited mitochondrial DNA synthesis.	[124]
	Activation of NR4A\DNA-PKcs\p53	Lactate accelerates vascular smooth muscle cell (VSMC) calcification by suppressing BCL2-interacting protein 3 (BNIP3)-mediated mitophagy. Lactate enhances mitochondrial fission through activation of the nuclear receptor subfamily 4 group A member 1 (NR4A1) pathway.	[125]
Retinal degeneration	Lactate-mediated regulation	Lactate activated autophagy by upregulating the ratio of LC3II/I, and increased formation of LC3 puncta and autophagic vacuole. Lactate prevented H_2_O_2_-induced mitochondrial fission and maintained mitochondrial function by alleviating H_2_O_2_-induced mitochondrial membrane potential disruption and intracellular ROS generation.	[126]
Maintain skeletal muscle function	Activation of SIRT1\PGC-1α	MCT1 deficiency leads to lactate accumulation in the cytoplasm, which, in turn, activates the SIRT1\PGC-1α signaling pathway to regulate mitochondrial biogenesis.	[127]
	Activation of Vps34	ULK1-mediated metabolic reprogramming leads to lactate accumulation and, in turn, lactated Vps34 increases lipid kinase activity to enhance mitochondrial autophagy and endolysosomal degradation.	[128]

## 7. Discussion and Outlook

This article offers an overview of the roles of lactate as a key signaling molecule involved in regulating post-translational modifications (PTMs) and influencing the epigenetic landscape. Aberrant histone lactylation has been increasingly linked to the pathogenesis of various diseases. Lactylation can alter the protein structure and function, impairing the activity of the mitochondrial respiratory chain and reducing energy production. It may also disrupt mitochondrial dynamics, including fusion and fission, thereby affecting mitochondrial quality and quantity.

Metabolic reprogramming is a hallmark of malignant transformation, primarily characterized by markedly increased glucose uptake to meet the heightened energy demands of tumor cells. This process is achieved through the tissue-specific overexpression of glucose transporters (GLUT family) and sodium-dependent glucose transporters (SGLTs), which facilitate glucose transport against concentration gradients and are closely associated with tumor invasion and metastatic phenotypes [129]. Targeting glucose transporters is already an established strategy for cancer therapy [130]. Therefore, future research should focus on elucidating the regulatory mechanisms of glucose transporters, optimizing targeted inhibitors, exploring combination therapies to effectively inhibit tumor metastasis.

The sustained activation of the glycolytic pathway leads to aberrant lactate overproduction. The underlying mechanisms involve not only the metabolic shift from mitochondrial oxidative phosphorylation to glycolysis, but also include compensatory mitochondrial functional adaptations. For instance, the p300-mediated acetylation of mitochondrial protein PDHX at the K488 site destabilizes the PDC, suppressing pyruvate conversion to acetyl-CoA and thereby exacerbating glycolysis-dependent lactate generation [131]. Notably, lactate accumulation drives pro-oncogenic gene expression via the induction of histone H3K56 lactylation, establishing a metabolic–epigenetic regulatory axis [132]. Concurrently, the dysregulated activity of mitochondrial inner membrane lactate transporters further disrupts the dynamic balance between glycolysis and OXPHOS, enhancing metabolic plasticity, allowing tumor cells to flexibly switch between energy production modes to evade therapeutic pressure. This metabolic heterogeneity poses therapeutic challenges, as monotherapies targeting rate-limiting glycolytic enzymes or mitochondrial electron transport chain inhibitors often induce drug resistance [133]. By contrast, combination strategies (glycolysis inhibitors with metformin) synergistically block the energy supply to improve efficacy. However, the causal relationship between metabolic reprogramming and mitochondrial dysfunction remains debated. Further studies are needed to determine whether mitochondrial OXPHOS abnormalities are primary drivers of carcinogenesis or secondary consequences of metabolic adaptation. Future research should prioritize the clinical translational potential of lactate metabolism inhibitors (LDHA inhibitors) and explore precision therapeutic strategies targeting lactate transporters and lactylation-mediated epigenetic regulatory networks.

As a byproduct of glycolytic metabolism, lactate has a dual role in the nervous system, primarily as an energy source for neurons to support synaptic function and neuroprotection through the mitochondrial TCA, especially in cerebral ischemia, where astrocytes attenuate neuronal damage by regulating lactate metabolism. However, its chronic accumulation disrupts the acid–base balance, inducing acidosis, mitochondrial oxidative stress, and glycolytic reprogramming, which exacerbates neurodegenerative disease processes.

The treatment of neurological diseases can be approached from three perspectives, including metabolic intervention, by targeting key enzymes of glycolysis or lactate transporter proteins in order to block pathological metabolic reprogramming, or the amelioration of mitochondrial oxidative stress by enhancing the expression of glycolytic enzymes. Next is epigenetic modulation, developing small-molecule drugs that specifically inhibit histone lactylation to reduce neuroinflammation. Alternatively, we can regulate related signal cascades, such as the AMPK/PINK1-Parkin pathway, to restore mitochondrial function, or LRP1 to regulate mitochondrial translocation from astrocytes to protect neuronal cells by restoring mitochondrial function. In the future, it will be necessary to deeply analyze the spatiotemporal dynamics of lactylation and its interactions with oxidative stress and the immune response, to develop site-specific inhibitors, as well as to explore the role of the metabolic–epigenetic network in the early stage of the disease, in order to identify new targets for the precision diagnosis and treatment of neurological diseases.

Existing studies on the relationship between metabolic reprogramming and mitochondrial dysfunction rely heavily on in vitro experiments—most commonly using cancer cell lines, primary neurons, and glial cell cultures. Such models cannot fully recapitulate the complex microenvironment observed in vivo; their high-glucose, hyperoxic, and hypoxic switch conditions and avascular milieu differ markedly from authentic tissue and, consequently, constrain the completeness of mechanistic insights. Thus, any therapeutic targets are identified in vitro mandate validation before advancing to the clinic. Moreover, the causal direction linking mitochondrial dysfunction and metabolic reprogramming remains unresolved. In oncological contexts, constitutive HIF-1α stabilization can initiate glycolytic reprogramming first, with ROS overproduction then culminating in secondary mitochondrial injury—suggesting that respiratory impairment may be a consequence rather than a trigger. Similarly, in neurodegenerative paradigms, microglial-glycolysis-derived lactate promotes histone lactylation, thereby upregulating pro-inflammatory cytokines and exacerbating mitochondrial dysfunction and neuroinflammation. These observations indicate a bidirectional or even cyclical causality between metabolic anomalies and mitochondrial damage, an issue that warrants further rigorous investigation.

Lactate and lactylation redefine the functional boundaries of metabolites in disease, as they are not merely byproducts of energy crises, but molecular switches that regulate the cellular fate. From the metabolic plasticity of tumors to the oxidative damage of neurons, from the apoptosis of cardiovascular cells to the functional decline in aging tissues, lactate and lactylation operate through a “metabolism–epigenetics–mitochondria” regulatory network, emerging as a potential therapeutic target across diseases. Future investigations should prioritize in vivo integrated approaches to delineate the causal loci of the lactate–mitochondria axis, while simultaneously exploring combinatorial intervention strategies targeting this axis to overcome the current constraints of single-target therapeutics. Critically, extrapolation boundaries for any in vitro findings must be explicitly circumscribed when translating to clinical contexts. Concurrently, elucidating the molecular mechanisms underlying lactylation remains indispensable, including the identification of specific modification sites, characterization of modifying enzymes, and discovery of de-modifying enzymes. These studies will enhance our understanding of lactate and lactylation in both physiological and pathological processes. Furthermore, a comprehensive exploration of how lactylation regulates mitochondrial functions, coupled with a comprehensive analysis of how mitochondrial dysfunction reciprocally influences lactylation levels, should be conducted to reveal the mechanisms underlying the interactions of lactate and lactylation with mitochondrial impairment. This research direction may facilitate the identification of novel therapeutic targets.

## Figures and Tables

**Table 1:** Lactylation targets with significant impact on mitochondrial function.

Target Proteins	Function	Effect of Lactylation	References
Histone H3K18	Regulation of gene transcription.	Acute myocardial infarction via H3K18la mediated apoptosis and mitophagy imbalance.	[18]
Histone H3K56	Regulates gene transcription and participates in DNA damage repair.	Mitochondrial pyruvate dehydrogenase inactivation shifts metabolism from OXPHOS to aerobic glycolysis, causing lactate accumulation that disrupts OXPHOS and enhances histone lactylation.	[46]
Histone H4K12	Regulation of glycolysis and inflammation-related gene expression, involved in metabolic reprogramming.	Enhancing microglial glycolysis elevates histone lactylation, thereby upregulating pyruvate kinase M2 expression.	[47]
ARF1	Small G proteins involved in vesicular trafficking and cytoskeletal dynamics.	K73la of ADP-ribosylation factor 1 (ARF1) modulates mitochondrial release and mitigates stroke-induced injury.	[48]
MDH2	Mitochondrial Malate dehydrogenase catalyzes the redox of malate and oxalacetic acid in the TCA cycle to produce NADH, which is involved in ATP synthesis.	Lactylation inhibits enzyme activity, reduces NADH production, and improves mitochondrial function in myocardial ischemia–reperfusion injury.	[44]
AK2	Mitochondrial Adenylate kinase 2 catalyzes the conversion of ADP to ATP and maintains the balance of mitochondrial energy metabolism.	K28la promotes the proliferation and metastasis of hepatocellular carcinoma cells and affects tumor progression by regulating mitochondrial metabolic pathways.	[49]
VDAC1	Mitochondrial voltage-dependent anion channel 1, which controls metabolite transmembrane transport, regulates mitochondrial apoptosis pathways.	Decreased lactylation levels at K20 and K266 impair channel activity, thereby participating in the regulation of mitochondrial apoptosis and neuronal death.	[50]
Fis1	Fis1 regulates mitochondrial fission by recruiting Drp1 to maintain mitochondrial morphology and function.	Elevated lactate at K20 promotes pathological mitochondrial excessive fission, triggering ATP depletion, mitochondrial ROS overproduction, and apoptotic signaling cascades.	[51]
ALDH2	Mitochondrial Acetaldehyde dehydrogenase 2, which metabolizes acetaldehyde and reactive aldehydes, is involved in anti-oxidative stress and nitroglycerin metabolism.	Decreased lactylation at the K52 reduces enzyme activity, leading to a decline in mitochondrial membrane potential.	[52]

## References

[1] Certo M., Tsai C.-H., Pucino V., Ho P.-C., Mauro C. (2021). Lactate Modulation of Immune Responses in Inflammatory versus Tumour Microenvironments. Nat. Rev. Immunol..

[2] Jiang H., Ren Y., Yu J., Hu S., Zhang J. (2023). Analysis of Lactate Metabolism-Related Genes and Their Association with Immune Infiltration in Septic Shock via Bioinformatics Method. Front. Genet..

[3] Barros L.F., Ruminot I., Martín A.S., Lerchundi R., Fernández-Moncada I., Baeza-Lehnert F. (2021). Aerobic Glycolysis in the Brain: Warburg and Crabtree Contra Pasteur. Neurochem. Res..

[4] Lee T.-Y. (2021). Lactate: A Multifunctional Signaling Molecule. Yeungnam Univ. J. Med..

[5] Zhang D., Tang Z., Huang H., Zhou G., Cui C., Weng Y., Liu W., Kim S., Lee S., Perez-Neut M. (2019). Metabolic Regulation of Gene Expression by Histone Lactylation. Nature.

[6] Flavahan W.A., Gaskell E., Bernstein B.E. (2017). Epigenetic Plasticity and the Hallmarks of Cancer. Science.

[7] Li X., Yang Y., Zhang B., Lin X., Fu X., An Y., Zou Y., Wang J.-X., Wang Z., Yu T. (2022). Lactate Metabolism in Human Health and Disease. Signal Transduct. Target. Ther..

[8] Li L., Chen K., Wang T., Wu Y., Xing G., Chen M., Hao Z., Zhang C., Zhang J., Ma B. (2020). Glis1 Facilitates Induction of Pluripotency via an Epigenome-Metabolome-Epigenome Signalling Cascade. Nat. Metab..

[9] Irizarry-Caro R.A., McDaniel M.M., Overcast G.R., Jain V.G., Troutman T.D., Pasare C. (2020). TLR Signaling Adapter BCAP Regulates Inflammatory to Reparatory Macrophage Transition by Promoting Histone Lactylation. Proc. Natl. Acad. Sci. USA.

[10] Yu J., Chai P., Xie M., Ge S., Ruan J., Fan X., Jia R. (2021). Histone Lactylation Drives Oncogenesis by Facilitating m6A Reader Protein YTHDF2 Expression in Ocular Melanoma. Genome Biol..

[11] Wan N., Wang N., Yu S., Zhang H., Tang S., Wang D., Lu W., Li H., Delafield D.G., Kong Y. (2022). Cyclic Immonium Ion of Lactyllysine Reveals Widespread Lactylation in the Human Proteome. Nat. Methods.

[12] Bonnay F., Veloso A., Steinmann V., Köcher T., Abdusselamoglu M.D., Bajaj S., Rivelles E., Landskron L., Esterbauer H., Zinzen R.P. (2020). Oxidative Metabolism Drives Immortalization of Neural Stem Cells during Tumorigenesis. Cell.

[13] Wang T., Sun F., Li C., Nan P., Song Y., Wan X., Mo H., Wang J., Zhou Y., Guo Y. (2023). MTA1, a Novel ATP Synthase Complex Modulator, Enhances Colon Cancer Liver Metastasis by Driving Mitochondrial Metabolism Reprogramming. Adv. Sci..

[14] Pan L., Feng F., Wu J., Fan S., Han J., Wang S., Yang L., Liu W., Wang C., Xu K. (2022). Demethylzeylasteral Targets Lactate by Inhibiting Histone Lactylation to Suppress the Tumorigenicity of Liver Cancer Stem Cells. Pharmacol. Res..

[15] Vander Linden C., Corbet C., Bastien E., Martherus R., Guilbaud C., Petit L., Wauthier L., Loriot A., De Smet C., Feron O. (2021). Therapy-Induced DNA Methylation Inactivates MCT1 and Renders Tumor Cells Vulnerable to MCT4 Inhibition. Cell Rep..

[16] He Y., Ji Z., Gong Y., Fan L., Xu P., Chen X., Miao J., Zhang K., Zhang W., Ma P. (2023). Numb/Parkin-Directed Mitochondrial Fitness Governs Cancer Cell Fate via Metabolic Regulation of Histone Lactylation. Cell Rep..

[17] Gu L., Ding X., Wang Y., Gu M., Zhang J., Yan S., Li N., Song Z., Yin J., Lu L. (2019). Spexin Alleviates Insulin Resistance and Inhibits Hepatic Gluconeogenesis via the FoxO1/PGC-1α Pathway in High-Fat-Diet-Induced Rats and Insulin Resistant Cells. Int. J. Biol. Sci..

[18] Silvis M.J.M., Demkes E.J., Fiolet A.T.L., Dekker M., Bosch L., van Hout G.P.J., Timmers L., de Kleijn D.P.V. (2021). Immunomodulation of the NLRP3 Inflammasome in Atherosclerosis, Coronary Artery Disease, and Acute Myocardial Infarction. J. Cardiovasc. Transl. Res..

[19] Calcinotto A., Kohli J., Zagato E., Pellegrini L., Demaria M., Alimonti A. (2019). Cellular Senescence: Aging, Cancer, and Injury. Physiol. Rev..

[20] Brooks G.A. (2020). Lactate as a Fulcrum of Metabolism. Redox Biol..

[21] Dou X., Fu Q., Long Q., Liu S., Zou Y., Fu D., Xu Q., Jiang Z., Ren X., Zhang G. (2023). PDK4-Dependent Hypercatabolism and Lactate Production of Senescent Cells Promotes Cancer Malignancy. Nat. Metab..

[22] Jha M.K., Lee I.-K., Suk K. (2016). Metabolic Reprogramming by the Pyruvate Dehydrogenase Kinase-Lactic Acid Axis: Linking Metabolism and Diverse Neuropathophysiologies. Neurosci. Biobehav. Rev..

[23] Felmlee M.A., Jones R.S., Rodriguez-Cruz V., Follman K.E., Morris M.E. (2020). Monocarboxylate Transporters (SLC16): Function, Regulation, and Role in Health and Disease. Pharmacol. Rev..

[24] Zhao R., Yi Y., Liu H., Xu J., Chen S., Wu D., Wang L., Li F. (2024). RHOF Promotes Snail1 Lactylation by Enhancing PKM2-Mediated Glycolysis to Induce Pancreatic Cancer Cell Endothelial-Mesenchymal Transition. Cancer Metab..

[25] Brown T.P., Ganapathy V. (2020). Lactate/GPR81 Signaling and Proton Motive Force in Cancer: Role in Angiogenesis, Immune Escape, Nutrition, and Warburg Phenomenon. Pharmacol. Ther..

[26] Moreno-Yruela C., Zhang D., Wei W., Bæk M., Liu W., Gao J., Danková D., Nielsen A.L., Bolding J.E., Yang L. (2022). Class I Histone Deacetylases (HDAC1-3) Are Histone Lysine Delactylases. Sci. Adv..

[27] Hagihara H., Shoji H., Otabi H., Toyoda A., Katoh K., Namihira M., Miyakawa T. (2021). Protein Lactylation Induced by Neural Excitation. Cell Rep..

[28] Gao X., Pang C., Fan Z., Wang Y., Duan Y., Zhan H. (2024). Regulation of Newly Identified Lysine Lactylation in Cancer. Cancer Lett..

[29] Gaffney D.O., Jennings E.Q., Anderson C.C., Marentette J.O., Shi T., Schou Oxvig A.-M., Streeter M.D., Johannsen M., Spiegel D.A., Chapman E. (2020). Non-Enzymatic Lysine Lactoylation of Glycolytic Enzymes. Cell Chem. Biol..

[30] Zhang D., Gao J., Zhu Z., Mao Q., Xu Z., Singh P.K., Rimayi C.C., Moreno-Yruela C., Xu S., Li G. (2025). Lysine L-Lactylation Is the Dominant Lactylation Isomer Induced by Glycolysis. Nat. Chem. Biol..

[31] Böttcher M., Baur R., Stoll A., Mackensen A., Mougiakakos D. (2020). Linking Immunoevasion and Metabolic Reprogramming in B-Cell-Derived Lymphomas. Front. Oncol..

[32] Katajisto P., Döhla J., Chaffer C.L., Pentinmikko N., Marjanovic N., Iqbal S., Zoncu R., Chen W., Weinberg R.A., Sabatini D.M. (2015). Stem Cells. Asymmetric Apportioning of Aged Mitochondria between Daughter Cells Is Required for Stemness. Science.

[33] Hinge A., He J., Bartram J., Javier J., Xu J., Fjellman E., Sesaki H., Li T., Yu J., Wunderlich M. (2020). Asymmetrically Segregated Mitochondria Provide Cellular Memory of Hematopoietic Stem Cell Replicative History and Drive HSC Attrition. Cell Stem Cell.

[34] Wang Q., Zhang Y., Yang C., Xiong H., Lin Y., Yao J., Li H., Xie L., Zhao W., Yao Y. (2010). Acetylation of Metabolic Enzymes Coordinates Carbon Source Utilization and Metabolic Flux. Science.

[35] Pongsuwan K., Kusirisin P., Narongkiattikhun P., Chattipakorn S.C., Chattipakorn N. (2022). Mitochondria and Vascular Calcification in Chronic Kidney Disease: Lessons Learned from the Past to Improve Future Therapy. J. Cell. Physiol..

[36] Zhou X., Xu S.-N., Yuan S.-T., Lei X., Sun X., Xing L., Li H.-J., He C.-X., Qin W., Zhao D. (2021). Multiple Functions of Autophagy in Vascular Calcification. Cell Biosci..

[37] Ma W., Jia K., Cheng H., Xu H., Li Z., Zhang H., Xie H., Sun H., Yi L., Chen Z. (2024). Orphan Nuclear Receptor NR4A3 Promotes Vascular Calcification via Histone Lactylation. Circ. Res..

[38] Shao G., Wang L., Wang X., Fu C. (2021). Apaf-1/Caspase-4 Pyroptosome: A Mediator of Mitochondrial Permeability Transition-Triggered Pyroptosis. Signal Transduct. Target. Ther..

[39] Mao Y., Zhang J., Zhou Q., He X., Zheng Z., Wei Y., Zhou K., Lin Y., Yu H., Zhang H. (2024). Hypoxia Induces Mitochondrial Protein Lactylation to Limit Oxidative Phosphorylation. Cell Res..

[40] Chen J., Zhang M., Liu Y., Zhao S., Wang Y., Wang M., Niu W., Jin F., Li Z. (2023). Histone Lactylation Driven by mROS-Mediated Glycolytic Shift Promotes Hypoxic Pulmonary Hypertension. J. Mol. Cell Biol..

[41] Caielli S., Cardenas J., de Jesus A.A., Baisch J., Walters L., Blanck J.P., Balasubramanian P., Stagnar C., Ohouo M., Hong S. (2021). Erythroid Mitochondrial Retention Triggers Myeloid-Dependent Type I Interferon in Human SLE. Cell.

[42] Li H., Boulougoura A., Endo Y., Tsokos G.C. (2022). Abnormalities of T Cells in Systemic Lupus Erythematosus: New Insights in Pathogenesis and Therapeutic Strategies. J. Autoimmun..

[43] Zhang J., Ji H., Liu M., Zheng M., Wen Z., Shen H. (2024). Mitochondrial DNA Programs Lactylation of cGAS to Induce IFN Responses in Patients with Systemic Lupus Erythematosus. J. Immunol..

[44] She H., Hu Y., Zhao G., Du Y., Wu Y., Chen W., Li Y., Wang Y., Tan L., Zhou Y. (2024). Dexmedetomidine Ameliorates Myocardial Ischemia-Reperfusion Injury by Inhibiting MDH2 Lactylation via Regulating Metabolic Reprogramming. Adv. Sci..

[45] Trefely S., Lovell C.D., Snyder N.W., Wellen K.E. (2020). Compartmentalised Acyl-CoA Metabolism and Roles in Chromatin Regulation. Mol. Metab..

[46] Son S.M., Park S.J., Breusegem S.Y., Larrieu D., Rubinsztein D.C. (2024). P300 Nucleocytoplasmic Shuttling Underlies mTORC1 Hyperactivation in Hutchinson-Gilford Progeria Syndrome. Nat. Cell Biol..

[47] Pan R.-Y., He L., Zhang J., Liu X., Liao Y., Gao J., Liao Y., Yan Y., Li Q., Zhou X. (2022). Positive Feedback Regulation of Microglial Glucose Metabolism by Histone H4 Lysine 12 Lactylation in Alzheimer’s Disease. Cell Metab..

[48] Zhou J., Zhang L., Peng J., Zhang X., Zhang F., Wu Y., Huang A., Du F., Liao Y., He Y. (2024). Astrocytic LRP1 Enables Mitochondria Transfer to Neurons and Mitigates Brain Ischemic Stroke by Suppressing ARF1 Lactylation. Cell Metab..

[49] Baumeister J., Chatain N., Hubrich A., Maié T., Costa I.G., Denecke B., Han L., Küstermann C., Sontag S., Seré K. (2020). Hypoxia-Inducible Factor 1 (HIF-1) Is a New Therapeutic Target in JAK2V617F-Positive Myeloproliferative Neoplasms. Leukemia.

[50] Yao Y., Bade R., Li G., Zhang A., Zhao H., Fan L., Zhu R., Yuan J. (2022). Global-Scale Profiling of Differential Expressed Lysine-Lactylated Proteins in the Cerebral Endothelium of Cerebral Ischemia–Reperfusion Injury Rats. Cell. Mol. Neurobiol..

[51] An S., Yao Y., Hu H., Wu J., Li J., Li L., Wu J., Sun M., Deng Z., Zhang Y. (2023). PDHA1 Hyperacetylation-Mediated Lactate Overproduction Promotes Sepsis-Induced Acute Kidney Injury via Fis1 Lactylation. Cell Death Dis..

[52] Li J., Shi X., Hou F., Luan X., Chen L. (2024). #1225 Lactate Aggravates Mitochondrial Dysfunction via ALDH2 Lactylation in Acute Kidney Injury. Nephrol. Dial. Transplant..

[53] Egger G., Liang G., Aparicio A., Jones P.A. (2004). Epigenetics in Human Disease and Prospects for Epigenetic Therapy. Nature.

[54] Zheng X., Chen J., Sun Y., Chen T., Wang J., Yu S. (2023). Mitochondria in Cancer Stem Cells: Achilles Heel or Hard Armor. Trends Cell Biol..

[55] Kopinski P.K., Singh L.N., Zhang S., Lott M.T., Wallace D.C. (2021). Mitochondrial DNA Variation and Cancer. Nat. Rev. Cancer.

[56] Chen Z., Liu M., Li L., Chen L. (2018). Involvement of the Warburg Effect in Non-Tumor Diseases Processes. J. Cell. Physiol..

[57] Cogliati S., Enriquez J.A., Scorrano L. (2016). Mitochondrial Cristae: Where Beauty Meets Functionality. Trends Biochem. Sci..

[58] Zhao Y., Wang A., Zou Y., Su N., Loscalzo J., Yang Y. (2016). In Vivo Monitoring of Cellular Energy Metabolism Using SoNar, a Highly Responsive Sensor for NAD(+)/NADH Redox State. Nat. Protoc..

[59] Jiang Z., Xiong N., Yan R., Li S.-T., Liu H., Mao Q., Sun Y., Shen S., Ye L., Gao P. (2025). PDHX Acetylation Facilitates Tumor Progression by Disrupting PDC Assembly and Activating Lactylation-Mediated Gene Expression. Protein Cell.

[60] Jiang H., Jin J., Duan Y., Xie Z., Li Y., Gao A., Gu M., Zhang X., Peng C., Xia C. (2019). Mitochondrial Uncoupling Coordinated With PDH Activation Safely Ameliorates Hyperglycemia via Promoting Glucose Oxidation. Diabetes.

[61] Zhu H., Chan K.T., Huang X., Cerra C., Blake S., Trigos A.S., Anderson D., Creek D.J., De Souza D.P., Wang X. (2022). Cystathionine-β-Synthase Is Essential for AKT-Induced Senescence and Suppresses the Development of Gastric Cancers with PI3K/AKT Activation. eLife.

[62] Lee Y.J., Shin K.J., Park S.-A., Park K.S., Park S., Heo K., Seo Y.-K., Noh D.-Y., Ryu S.H., Suh P.-G. (2016). G-Protein-Coupled Receptor 81 Promotes a Malignant Phenotype in Breast Cancer through Angiogenic Factor Secretion. Oncotarget.

[63] Sun L., Zhang Y., Yang B., Sun S., Zhang P., Luo Z., Feng T., Cui Z., Zhu T., Li Y. (2023). Lactylation of METTL16 Promotes Cuproptosis via m6A-Modification on FDX1 mRNA in Gastric Cancer. Nat. Commun..

[64] Koppenol W.H., Bounds P.L., Dang C.V. (2011). Otto Warburg’s Contributions to Current Concepts of Cancer Metabolism. Nat. Rev. Cancer.

[65] Yang J., Luo L., Zhao C., Li X., Wang Z., Zeng Z., Yang X., Zheng X., Jie H., Kang L. (2022). A Positive Feedback Loop between Inactive VHL-Triggered Histone Lactylation and PDGFRβ Signaling Drives Clear Cell Renal Cell Carcinoma Progression. Int. J. Biol. Sci..

[66] Qiao K., Chen C., Liu H., Qin Y., Liu H. (2021). Pinin Induces Epithelial-to-Mesenchymal Transition in Hepatocellular Carcinoma by Regulating m6A Modification. J. Oncol..

[67] Yue Q., Wang Z., Shen Y., Lan Y., Zhong X., Luo X., Yang T., Zhang M., Zuo B., Zeng T. (2024). Histone H3K9 Lactylation Confers Temozolomide Resistance in Glioblastoma via LUC7L2-Mediated MLH1 Intron Retention. Adv. Sci..

[68] Luo Y., Yang Z., Yu Y., Zhang P. (2022). HIF1α Lactylation Enhances KIAA1199 Transcription to Promote Angiogenesis and Vasculogenic Mimicry in Prostate Cancer. Int. J. Biol. Macromol..

[69] Yang Z., Yan C., Ma J., Peng P., Ren X., Cai S., Shen X., Wu Y., Zhang S., Wang X. (2023). Lactylome Analysis Suggests Lactylation-Dependent Mechanisms of Metabolic Adaptation in Hepatocellular Carcinoma. Nat. Metab..

[70] Kong D., Liu C., Miao X., Wang Y., Ding X., Gong W. (2020). Current Statuses of Molecular Targeted and Immune Checkpoint Therapies in Hepatocellular Carcinoma. Am. J. Cancer Res..

[71] Jiang J., Huang D., Jiang Y., Hou J., Tian M., Li J., Sun L., Zhang Y., Zhang T., Li Z. (2021). Lactate Modulates Cellular Metabolism Through Histone Lactylation-Mediated Gene Expression in Non-Small Cell Lung Cancer. Front. Oncol..

[72] Jia L., Liao M., Mou A., Zheng Q., Yang W., Yu Z., Cui Y., Xia X., Qin Y., Chen M. (2021). Rheb-Regulated Mitochondrial Pyruvate Metabolism of Schwann Cells Linked to Axon Stability. Dev. Cell.

[73] Huang M., Wu Y., Cheng L., Fu L., Yan H., Ru H., Mo X., Yan L., Su Z. (2023). Multi-Omics Analyses of Glucose Metabolic Reprogramming in Colorectal Cancer. Front. Immunol..

[74] Wei Y., Miao Q., Zhang Q., Mao S., Li M., Xu X., Xia X., Wei K., Fan Y., Zheng X. (2023). Aerobic Glycolysis Is the Predominant Means of Glucose Metabolism in Neuronal Somata, Which Protects against Oxidative Damage. Nat. Neurosci..

[75] Calsolaro V., Edison P. (2016). Neuroinflammation in Alzheimer’s Disease: Current Evidence and Future Directions. Alzheimers Dement..

[76] Yoon N.A., Jin S., Kim J.D., Liu Z.W., Sun Q., Cardone R., Kibbey R., Diano S. (2022). UCP2-Dependent Redox Sensing in POMC Neurons Regulates Feeding. Cell Rep..

[77] Wei L., Yang X., Wang J., Wang Z., Wang Q., Ding Y., Yu A. (2023). H3K18 Lactylation of Senescent Microglia Potentiates Brain Aging and Alzheimer’s Disease through the NFκB Signaling Pathway. J. Neuroinflamm..

[78] Qin Q., Wang D., Qu Y., Li J., An K., Mao Z., Li J., Xiong Y., Min Z., Xue Z. (2025). Enhanced Glycolysis-Derived Lactate Promotes Microglial Activation in Parkinson’s Disease via Histone Lactylation. NPJ Park. Dis..

[79] Wang Z., Hao D., Zhao S., Zhang Z., Zeng Z., Wang X. (2023). Lactate and Lactylation: Clinical Applications of Routine Carbon Source and Novel Modification in Human Diseases. Mol. Cell. Proteom..

[80] Xue X., Wang H., Su J. (2020). Inhibition of MiR-122 Decreases Cerebral Ischemia-Reperfusion Injury by Upregulating DJ-1-Phosphatase and Tensin Homologue Deleted on Chromosome 10 (PTEN)/Phosphonosinol-3 Kinase (PI3K)/AKT. Med. Sci. Monit..

[81] Liu C., Wang G., Han W., Tian Q., Li M. (2023). Ferroptosis: A Potential Therapeutic Target for Stroke. Neural Regen. Res..

[82] Lv Y., Shao G., Zhang Q., Wang X., Meng Y., Wang L., Huang F., Yang T., Jin Y., Fu C. (2019). The Antimicrobial Peptide PFR Induces Necroptosis Mediated by ER Stress and Elevated Cytoplasmic Calcium and Mitochondrial ROS Levels: Cooperation with Ara-C to Act against Acute Myeloid Leukemia. Signal Transduct. Target. Ther..

[83] Nagase M., Takahashi Y., Watabe A.M., Kubo Y., Kato F. (2014). On-Site Energy Supply at Synapses through Monocarboxylate Transporters Maintains Excitatory Synaptic Transmission. J. Neurosci..

[84] Wang Y., Liu H.-H., Cao Y.-T., Zhang L.-L., Huang F., Yi C. (2020). The Role of Mitochondrial Dynamics and Mitophagy in Carcinogenesis, Metastasis and Therapy. Front. Cell Dev. Biol..

[85] Guerreiro S., Privat A.-L., Bressac L., Toulorge D. (2020). CD38 in Neurodegeneration and Neuroinflammation. Cells.

[86] Hinarejos I., Machuca-Arellano C., Sancho P., Espinós C. (2020). Mitochondrial Dysfunction, Oxidative Stress and Neuroinflammation in Neurodegeneration with Brain Iron Accumulation (NBIA). Antioxidants.

[87] Magistretti P.J. (2011). Neuron–Glia Metabolic Coupling and Plasticity. Exp. Physiol..

[88] Brooks G.A., Arevalo J.A., Osmond A.D., Leija R.G., Curl C.C., Tovar A.P. (2022). Lactate in Contemporary Biology: A Phoenix Risen. J. Physiol..

[89] Cai M., Wang H., Song H., Yang R., Wang L., Xue X., Sun W., Hu J. (2022). Lactate Is Answerable for Brain Function and Treating Brain Diseases: Energy Substrates and Signal Molecule. Front. Nutr..

[90] Saab A.S., Tzvetavona I.D., Trevisiol A., Baltan S., Dibaj P., Kusch K., Möbius W., Goetze B., Jahn H.M., Huang W. (2016). Oligodendroglial NMDA Receptors Regulate Glucose Import and Axonal Energy Metabolism. Neuron.

[91] Hollnagel J.-O., Cesetti T., Schneider J., Vazetdinova A., Valiullina-Rakhmatullina F., Lewen A., Rozov A., Kann O. (2020). Lactate Attenuates Synaptic Transmission and Affects Brain Rhythms Featuring High Energy Expenditure. iScience.

[92] Hsieh C.-F., Liu C.-K., Lee C.-T., Yu L.-E., Wang J.-Y. (2019). Acute Glucose Fluctuation Impacts Microglial Activity, Leading to Inflammatory Activation or Self-Degradation. Sci. Rep..

[93] Ishihara Y., Itoh K. (2023). Microglial Inflammatory Reactions Regulated by Oxidative Stress. J. Clin. Biochem. Nutr..

[94] Hu Y., Mai W., Chen L., Cao K., Zhang B., Zhang Z., Liu Y., Lou H., Duan S., Gao Z. (2020). mTOR-Mediated Metabolic Reprogramming Shapes Distinct Microglia Functions in Response to Lipopolysaccharide and ATP. Glia.

[95] Mathys H., Davila-Velderrain J., Peng Z., Gao F., Mohammadi S., Young J.Z., Menon M., He L., Abdurrob F., Jiang X. (2019). Single-Cell Transcriptomic Analysis of Alzheimer’s Disease. Nature.

[96] Andersen J.V., Markussen K.H., Jakobsen E., Schousboe A., Waagepetersen H.S., Rosenberg P.A., Aldana B.I. (2021). Glutamate Metabolism and Recycling at the Excitatory Synapse in Health and Neurodegeneration. Neuropharmacology.

[97] Halim N.D., Mcfate T., Mohyeldin A., Okagaki P., Korotchkina L.G., Patel M.S., Jeoung N.H., Harris R.A., Schell M.J., Verma A. (2010). Phosphorylation Status of Pyruvate Dehydrogenase Distinguishes Metabolic Phenotypes of Cultured Rat Brain Astrocytes and Neurons. Glia.

[98] Vlassenko A.G., Gordon B.A., Goyal M.S., Su Y., Blazey T.M., Durbin T.J., Couture L.E., Christensen J.J., Jafri H., Morris J.C. (2018). Aerobic Glycolysis and Tau Deposition in Preclinical Alzheimer’s Disease. Neurobiol. Aging.

[99] Fu J., Lu Z.-T., Wu G., Yang Z.-C., Wu X., Wang D., Nie Z.-M., Sheng Q. (2024). Gastrodia Elata Specific miRNA Attenuates Neuroinflammation via Modulating NF-κB Signaling Pathway. Int. J. Neurosci..

[100] Tauffenberger A., Fiumelli H., Almustafa S., Magistretti P.J. (2019). Lactate and Pyruvate Promote Oxidative Stress Resistance through Hormetic ROS Signaling. Cell Death Dis..

[101] Hadzic A., Nguyen T.D., Hosoyamada M., Tomioka N.H., Bergersen L.H., Storm-Mathisen J., Morland C. (2020). The Lactate Receptor HCA1 Is Present in the Choroid Plexus, the Tela Choroidea, and the Neuroepithelial Lining of the Dorsal Part of the Third Ventricle. Int. J. Mol. Sci..

[102] Zheng J., Xie Y., Ren L., Qi L., Wu L., Pan X., Zhou J., Chen Z., Liu L. (2021). GLP-1 Improves the Supportive Ability of Astrocytes to Neurons by Promoting Aerobic Glycolysis in Alzheimer’s Disease. Mol. Metab..

[103] Kettunen M.I., Hu D., Witney T.H., McLaughlin R., Gallagher F.A., Bohndiek S.E., Day S.E., Brindle K.M. (2010). Magnetization Transfer Measurements of Exchange between Hyperpolarized [1-13C]Pyruvate and [1-13C]Lactate in a Murine Lymphoma. Magn. Reson. Med..

[104] Pajares M., Rojo I.A., Manda G., Boscá L., Cuadrado A. (2020). Inflammation in Parkinson’s Disease: Mechanisms and Therapeutic Implications. Cells.

[105] Xi Y., Tao K., Wen X., Feng D., Mai Z., Ding H., Mao H., Wang M., Yang Q., Xiang J. (2025). SIRT3-Mediated Deacetylation of DRP1K711 Prevents Mitochondrial Dysfunction in Parkinson’s Disease. Adv. Sci..

[106] Li J., Chen L., Qin Q., Wang D., Zhao J., Gao H., Yuan X., Zhang J., Zou Y., Mao Z. (2022). Upregulated Hexokinase 2 Expression Induces the Apoptosis of Dopaminergic Neurons by Promoting Lactate Production in Parkinson’s Disease. Neurobiol. Dis..

[107] Chen X.-L., Wang Y., Peng W.-W., Zheng Y.-J., Zhang T.-N., Wang P.-J., Huang J.-D., Zeng Q.-Y. (2018). Effects of Interleukin-6 and IL-6/AMPK Signaling Pathway on Mitochondrial Biogenesis and Astrocytes Viability under Experimental Septic Condition. Int. Immunopharmacol..

[108] Li M., Li M., Lin Z., Zhuang Y., Wang H., Jia J., Lu Y., Wang Z., Zou H., Zhao H. (2024). Buyang Huanwu Decoction Promotes Neurovascular Remodeling by Modulating Astrocyte and Microglia Polarization in Ischemic Stroke Rats. J. Ethnopharmacol..

[109] Chen C., Chen Y., Liu T., Song D., Ma D., Cheng O. (2022). Dexmedetomidine Can Enhance PINK1/Parkin-Mediated Mitophagy in MPTP-Induced PD Mice Model by Activating AMPK. Oxidative Med. Cell. Longev..

[110] Obsilova V., Obsil T. (2022). Structural Insights into the Functional Roles of 14-3-3 Proteins. Front. Mol. Biosci..

[111] Gao W., Yang J., Liu W., Wang Y., Shao F. (2016). Site-Specific Phosphorylation and Microtubule Dynamics Control Pyrin Inflammasome Activation. Proc. Natl. Acad. Sci. USA.

[112] Structure of Human PINK1 at a Mitochondrial TOM-VDAC Array. https://www.science.org/doi/10.1126/science.adu6445.

[113] Li X., Fu X., Li H., Gao Y., Wang W., Shen Y. (2023). Leptin Differentially Regulate Cell Apoptosis and Cycle by Histone Acetylation in Tibial and Vertebral Epiphyseal Plates. Cell Biol. Int..

[114] Susser L.I., Nguyen M.-A., Geoffrion M., Emerton C., Ouimet M., Khacho M., Rayner K.J. (2023). Mitochondrial Fragmentation Promotes Inflammation Resolution Responses in Macrophages via Histone Lactylation. Mol. Cell. Biol..

[115] Wang K., Liu H., Hu Q., Wang L., Liu J., Zheng Z., Zhang W., Ren J., Zhu F., Liu G.-H. (2022). Epigenetic Regulation of Aging: Implications for Interventions of Aging and Diseases. Signal Transduct. Target. Ther..

[116] Im C.-N., Seo J.-S. (2014). Overexpression of Tumor Necrosis Factor Receptor-Associated Protein 1 (TRAP1), Leads to Mitochondrial Aberrations in Mouse Fibroblast NIH/3T3 Cells. BMB Rep..

[117] Li X., Chen M., Chen X., He X., Li X., Wei H., Tan Y., Min J., Azam T., Xue M. (2024). TRAP1 Drives Smooth Muscle Cell Senescence and Promotes Atherosclerosis via HDAC3-Primed Histone H4 Lysine 12 Lactylation. Eur. Heart J..

[118] Lv M., Zhou W., Hao Y., Li F., Zhang H., Yao X., Shi Y., Zhang L. (2024). Structural Insights into the Specific Recognition of Mitochondrial Ribosome-Binding Factor hsRBFA and 12 S rRNA by Methyltransferase METTL15. Cell Discov..

[119] Hong W., Zeng X., Wang H., Tan X., Tian Y., Hu H., Ashrafizadeh M., Sethi G., Huang H., Duan C. (2024). PGC-1α Loss Promotes Mitochondrial Protein Lactylation in Acetaminophen-Induced Liver Injury via the LDHB-Lactate Axis. Pharmacol. Res..

[120] Li J., Shi X., Xu J., Wang K., Hou F., Luan X., Chen L. (2025). Aldehyde Dehydrogenase 2 Lactylation Aggravates Mitochondrial Dysfunction by Disrupting PHB2 Mediated Mitophagy in Acute Kidney Injury. Adv. Sci..

[121] Ye F., Huang J., Wang H., Luo C., Zhao K. (2019). Targeting Epigenetic Machinery: Emerging Novel Allosteric Inhibitors. Pharmacol. Ther..

[122] Yuan J., Yang M., Wu Z., Wu J., Zheng K., Wang J., Zeng Q., Chen M., Lv T., Shi Y. (2025). The Lactate-Primed KAT8-PCK2 Axis Exacerbates Hepatic Ferroptosis During Ischemia/Reperfusion Injury by Reprogramming OXSM-Dependent Mitochondrial Fatty Acid Synthesis. Adv. Sci..

[123] Sun Z., Ji Z., Meng H., He W., Li B., Pan X., Zhou Y., Yu G. (2024). Lactate Facilitated Mitochondrial Fission-Derived ROS to Promote Pulmonary Fibrosis via ERK/DRP-1 Signaling. J. Transl. Med..

[124] Zhu Y., Zhang J.-L., Yan X.-J., Ji Y., Wang F.-F. (2023). Exploring a New Mechanism between Lactate and VSMC Calcification: PARP1/POLG/UCP2 Signaling Pathway and Imbalance of Mitochondrial Homeostasis. Cell Death Dis..

[125] Zhu Y., Ji J.-J., Yang R., Han X.-Q., Sun X.-J., Ma W.-Q., Liu N.-F. (2019). Lactate Accelerates Calcification in VSMCs through Suppression of BNIP3-Mediated Mitophagy. Cell. Signal..

[126] Zou G.-P., Wang T., Xiao J.-X., Wang X.-Y., Jiang L.-P., Tou F.-F., Chen Z.-P., Qu X.-H., Han X.-J. (2023). Lactate Protects against Oxidative Stress-Induced Retinal Degeneration by Activating Autophagy. Free. Radic. Biol. Med..

[127] Zhang L., Xin C., Wang S., Zhuo S., Zhu J., Li Z., Liu Y., Yang L., Chen Y. (2024). Lactate Transported by MCT1 Plays an Active Role in Promoting Mitochondrial Biogenesis and Enhancing TCA Flux in Skeletal Muscle. Sci. Adv..

[128] Jia M., Yue X., Sun W., Zhou Q., Chang C., Gong W., Feng J., Li X., Zhan R., Mo K. (2023). ULK1-Mediated Metabolic Reprogramming Regulates Vps34 Lipid Kinase Activity by Its Lactylation. Sci. Adv..

[129] Wood I.S., Trayhurn P. (2003). Glucose Transporters (GLUT and SGLT): Expanded Families of Sugar Transport Proteins. Br. J. Nutr..

[130] Barbosa A.M., Martel F. (2020). Targeting Glucose Transporters for Breast Cancer Therapy: The Effect of Natural and Synthetic Compounds. Cancers.

[131] Senyilmaz D., Teleman A.A. (2015). Chicken or the Egg: Warburg Effect and Mitochondrial Dysfunction. F1000Prime Rep..

[132] Kee H.J., Cheong J.-H. (2014). Tumor Bioenergetics: An Emerging Avenue for Cancer Metabolism Targeted Therapy. BMB Rep..

[133] Zhou F., Zhu H., Fu C. (2022). Editorial: Clinical Therapeutic Development Against Cancers Resistant to Targeted Therapies. Front. Pharmacol..

